# Gender Disparities in Endowed Professorships Within Orthopaedic Surgery

**DOI:** 10.7759/cureus.55180

**Published:** 2024-02-28

**Authors:** Alicia M Asturias, Aboubacar Wague, Leena A Feeley, Carlin Senter, Nirav Pandya, Brian T Feeley

**Affiliations:** 1 Orthopaedic Surgery, University of California San Francisco, San Francisco, USA

**Keywords:** women in medicine, women in orthopaedic surgery, women in surgery, orthopedic surgeon, equity and inclusion, inclusion and diversity, underrepresented gender and racial minority, gender equity

## Abstract

Background

Orthopaedic surgery has the lowest number of full-time faculty positions held by women, at 19%, with endowed chairs among the most coveted and advantageous. We examined the characteristics of endowed professors from the US top 100 orthopaedic academic centers and highest-funded musculoskeletal (MSK) researchers to determine if gender is associated with endowed professorship. Additionally, we sought to determine if gender is associated with increased NIH funding for top-performing musculoskeletal researchers.

Methods

Our primary study group included the top 100 orthopaedic academic centers defined by US News World Report and Doximity’s rankings. Our secondary study group examined the top MSK researchers, defined as principal investigators, who received >$400,000 in annual NIH funding from 2018 to 2021. Orthopaedic departments included MSK researchers and subspecialties within orthopaedics and medicine. Publicly available sources were used to compile institutional, gender, H-index, citation number, and subspecialty data on endowed professors; statistical comparisons were calculated.

Results

Within the top 100 orthopaedic academic departments, 4674 faculty were identified. Seven hundred and thirty-three (15.68%) were identified as women, 3941 as men (84.32%). One hundred and ninety-four held endowed professorships; 13 were awarded to women (6.7%), and 185 (95.3%) were awarded to men, with a significant odds ratio (OR) of 2.95, favoring men. For MSK researchers, the OR increases to 11.4. Arthroplasty and sports had the highest numbers of endowments. Significant differences in H-index, publications, and graduation year were identified between men and women for top MSK researchers and orthopaedic-trained surgeons; however, these differences disappeared when considering heterogenous orthopaedic departments that included medicine subspecialties, plastic surgery, hand surgery, and neurosurgery. Additional gender differences were observed in endowment names, with awards commemorating 51.5% men, 7.2% women, and 34% families or groups.

Conclusion

Gender inequities at the endowment level are substantial, and there are very few women in musculoskeletal medicine to achieve endowments. Differences in H-index, publications, and graduation year between men and women MSK researchers and orthopaedic-trained surgeons, but not combined orthopaedic, PM&R, and medical subspecialty departments, suggest unique challenges in orthopaedic surgery environments and histories that may contribute to endowment disparity. Gender was not found to be associated with funding bias for top-performing musculoskeletal researchers.

## Introduction

Musculoskeletal problems are among the most common chief concerns seen across healthcare fields, with a growing body of resources and research devoted to the care of these issues across all genders and age groups. Despite this, orthopaedic surgery departments have struggled to represent this same level of diversity within their faculty, with practicing orthopaedic women representing less than 8% of orthopaedic surgeons and even less still for other underrepresented communities [1,2]. It is well documented that improved provider diversity improves care when it is delivered in a culturally competent manner [3]. There have been concerted efforts to address this discrepancy at the pre-clinical, medical school, residency, and faculty levels, including mandatory musculoskeletal (MSK) curricula at medical schools and recruitment programs such as Nth Dimensions and the Perry Initiative [1,3-5]. Programs with more female faculty members and women in leadership have more female residents, and applicants rank the number of women within a department in the Top 5 reasons for choosing a particular program [4,6-8]. Thus, representation matters, and it matters at the leadership level [4]. However, progress is slow. Van Heest et al., in their 15-year report on the Uneven Distribution of Women in Orthopaedic Training project, found that at the current rate of improvement, orthopaedics will not achieve the goal of 30% diversity in orthopaedic training until 2060 [7,9].

While many studies have commented on disparities at the chair, department, and professorship levels [4,6-8,10-13], no prior studies have examined the disparate proportions of men and women who currently have endowment positions within orthopaedic surgery. Since the early 1900s, endowments in academia have represented autonomy, stability, and a capitalistic advantage for recipients, spurring competition between the wealthiest universities [14]. Gold et al. noted gender disparities at the endowment level within their subspecialties in internal medicine, a field in which the gender gap is considerably narrower. They concluded that within top-tier US internal medicine departments, female full-time professors are less likely to hold endowed chairs than their male counterparts. Gender differences persisted even after adjustments for factors such as academic performance [15]. 

Our study looks to examine the gender distribution of endowed chairs in orthopaedic surgery and MSK research and to determine if gender is an independent variable in the acquisition of an endowed professorship. Additionally, we look to examine whether or not there are gender differences in NIH funding for MSK researchers. A gender gap at this level would represent a new glass ceiling for female orthopaedic leadership and thus warrants analysis. 

This article was presented at the Orthopaedic Research Society Annual Meeting in the resident and fellow research section on February 12th, 2023. 

## Materials and methods

Our primary study group represented orthopaedic surgery faculty from the Top 100 Academic Residency Departments in the United States based on the US News World Report and Doximity’s orthopaedic surgery rank lists [25]. A secondary study group, including the Top MSK researchers, defined principal investigators as the ones who received >$400,000 in annual NIH funding in 2018, 2019, 2020, and 2021 [26]. Focusing on the top 100 academic orthopaedic departments allowed our study population to reach nearly 5000 physicians, providing sufficient power for our analysis. Similarly, the decision to focus on MSK researchers awarded >$400,000 in NIH funding provided sufficient data to reach an appropriate power analysis and appropriately reflected top-tier researchers with objective NIH data. Orthopaedic departments included MSK researchers and clinical subspecialties within orthopaedics and MSK medicine, as this information was readily available and utilized the same data available to patients when choosing providers. Ancillary providers and physical therapists were not included in the primary faculty group.

For each cohort, publicly available sources, including Scopus (Elsevier), institutional websites, and state licensing boards, were used to compile institutional, gender, H-index, citation number, and subspecialty data for each endowed professor. Name and faculty photo were used to estimate gender if not otherwise specified; non-binary gender was not included for this study. Graduation year was not reliably reported to be used as a covariable for the heterogeneous departmental cohort but was accessible for the endowed MD and MSK researchers cohort. Academic rank was excluded, given the heterogeneity of titles between programs (Assistant Professor, Clinical Faculty, Co-Chair, Vice-Chair, Division Chief, Departmental Chief, etc.). For the MSK research group, this multivariable data was obtained for researchers both with and without endowments to allow for multivariate regression analysis. For the orthopaedic department group, only gender data was obtained for individuals with and without endowments, while multivariate data was obtained for endowment awardees. Data was obtained in real-time using publicly available information during 2022. Given no identifiable private information was available about the participants included, the conducting institution determined informed consent or IRB regulation was not required; however, where public data was conflicting, individuals were contacted to confirm the accuracy of the available information. The number of contacted individuals was less than 10. Means were calculated and compared using two-tailed T-tests and Pearson chi-square tests when appropriate. Multivariate logistic regression was calculated for the MSK research cohort using Prism (Ver 9.4.0).

## Results

**Figure 1:**
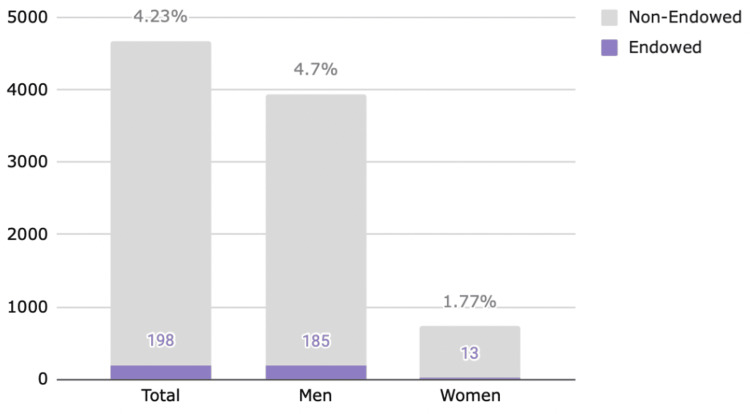
Endowment by gender

In our primary study group, 4674 clinical faculty were identified within the Top 100 orthopaedic academic departments. Seven hundred and thirty-three (15.68%) were identified as women, 3941 as men (84.32%). The proportion of women was notably higher than estimations for orthopaedic surgery alone, as our cohort included medical subspecialties such as rheumatology and sports medicine [6]. Of the total of 4674, 198 held titles of endowed professorships. Thirteen (6.5%) endowed professorships were awarded to women, and 185 (93%.4) were awarded to men, with a significant odds ratio of 2.95 favoring men (Table 1, Figure 1). 

**Table 1:** Gender disparities in musculoskeletal researchers and MDs *significance calculated with T-test, **significance calculated with Chi-squared test

Characteristic	Endowed	Non-Endowed	Total	95% Confidence Interval
Total	198	4480	4674	
Women	13	720	733	
Men	185	3756	3941	
Odds Ratio M:F Endowments		2.95*		1.6 - 5.33

One hundred and sixty-nine of the 198 total endowments were awarded to orthopaedic-trained surgeons, eight (4.1%) of whom were women, and the remaining 161 (81.3%) were men. In this group, there were differences between the mean H-index, citation, and graduation year between men and women orthopaedic endowment awardees (Table 2). 

**Table 2:** Characteristics of endowed orthopaedic-trained MDs **proportion significance calculated with 96% CI compared to all other groups distribution -1.96 - +1.96 *p-value with significance 0.05

Characteristic	Women	Men	Total	P-Value or Z-Score
Orthopaedic Endowments	8	161	169.00	-16.64**
MD Only	8	151	159.00	-3.98**
MD/PhD	0	10	10.00	-0.75
Mean H-Index	10.50	32.01	30.98	0.00*
Mean Publications	36.90	141.90	144.40	0.042*
Graduation Year	2004	1996	1996	0.001*

**Figure 2:**
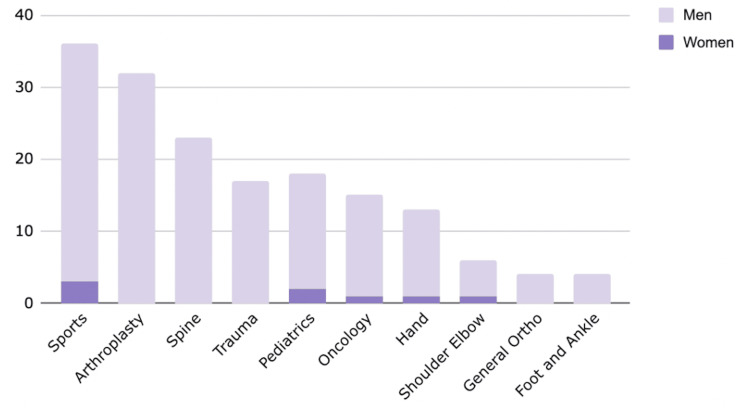
Endowment by specialty

However, when performing the same analysis using the heterogenous cohort of medicine subspecialties, plastics hand, neurosurgery spine, and MSK researchers, there are no significant differences in H-index, publications, or graduation year, suggesting that there are unique environmental challenges in orthopaedic surgery that continue to contribute to the disparity, for which senior orthopaedic men may be a modifier. Arthroplasty and sports held the highest number of endowments, comprising 19% and 21.3%, respectively, with only three female recipients represented between the two (Figure 2).

**Figure 3:**
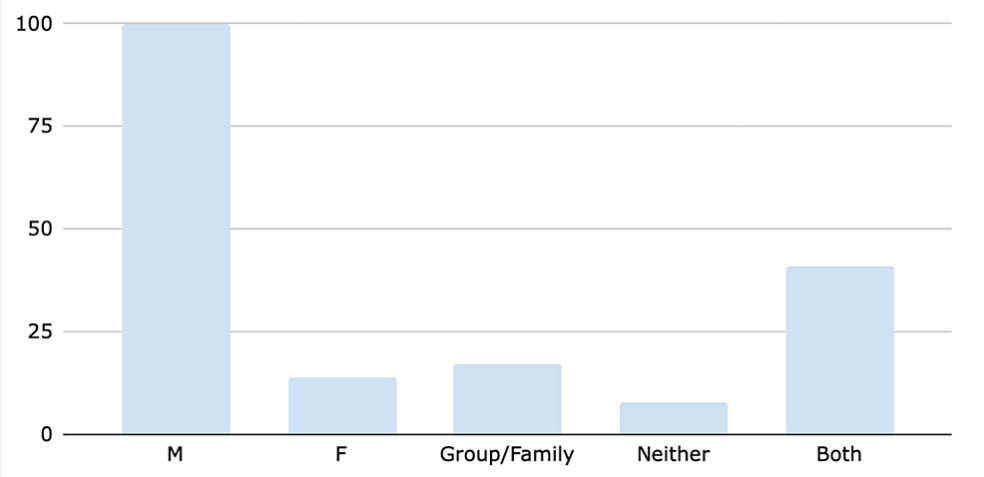
Endowment donor name/gender

Further analysis revealed gender differences in endowment names, with awards commemorating (51.5%) men, (7.2%) women, and (34%) families or groups (Figure 3). 

In our secondary study group, 137 top NIH-funded MSK principal investigators were identified. 29% (40) were women, and 71% (97) were men. Twenty-three researchers (16.8%) were awarded named endowed professorships, 22 of which were awarded to men, and only one was awarded to a female researcher (Table 3). In this secondary study group of MSK researchers, the percentage of women is 29%, and only one holds the title of endowment (2.5%), whereas of the 71% male MSK researchers, 22 hold the title of endowment (22.6%). 

**Table 3:** Characteristics of total musculoskeletal principal investigators (PIs) *significance for p-value set at 0.05 with a two-tailed t-test **significance for p-value set at 0.05 with Chi-square test

Characteristic	Women	Men	Total	95% Confidence Interval or P-Value
All PIs	40	97	137	
Endowments	1	22	23	
Odds Ratio M:W	11.44			1.4 - 88.1
MD Only	0	9	9	
PhD Only	40	79	118	
MD/PhD	1	8	9	
Average NIH Funding	$701,999.75	$952,515.59	$816,506.10	0.37
H-Index	27.85	40.09	36.52	0.001*
H-Index Range	9-65	11-104	9-104	
Number Publications	75.20	142.81	123.07	0.001*
Graduate Year	2002	1999	2000	0.07

Of note, the first endowment of a woman was at a funding ranking of 137. Of those awarded, 15 have PhDs alone; one has an MD alone; and six have both an MD and PhD, including the female endowed chair. Of the seven MSK researchers with an MD and endowment, five are orthopaedically trained surgeons, and the remaining two are internal medicine trained, including the female endowed chair. All 40 of the female principal investigators (PIs) have PhDs (Table 3). 

For the MSK research group, on multivariate analysis, when controlling for H-index, year of graduation, publication number, and degree, gender was an independent predictor of endowment (p <0.001). Endowed chairs received degrees earlier than their non-endowed counterparts, graduating in 1995 versus 2001 (Table 3). Endowed chairs had a significantly higher average H-index compared to our non-endowed cohort, with H-indices of 52.8 versus 33.1, respectively (p = 0.001, Table 3). Additionally, there were significant differences in H-index and publications between genders, with women reporting 27.85 and men reporting 40.09, respectively (Table 3). There was no significant difference between the average NIH funding between endowed women ($701,999.75) and men ($952,515.59) in the research cohort (Table 3); however, there were differences between endowed and non-endowed PIs regardless of gender (Table 4). 

**Table 4:** Characteristics of musculoskeletal professors by endowment

Characteristic	Endowed	Not Endowed	Total	P-Value
Average Funding	$1,788,106	$696,031	$816,506	0.001*
H-Index	53.39	33.11	36.52	<0.000*
H-Index Range	59-101	9-104	9-104	
Number Publications	225.17	123.07	123.07	<0.000*
Graduation Year	1996	2001	2000	0.018*

## Discussion

In this study, we demonstrated that endowments for women in orthopaedic surgery and MSK research are rare; in fact, there are only 13. Removing full-time researchers and medical sub-specialists within orthopaedic surgery, that number shrinks further still to only eight. Sports has the highest number of women represented at three; however, this number remains low given the percentage of women in the field, which has been reported at 16.3% [13]. Multivariate analysis suggested that gender was predictive of endowment for MSK researchers, and male MSK researchers are 11 times more likely to achieve endowment than their female counterparts. For orthopaedic departments, men are nearly three times more likely to obtain endowments than women, a number likely underestimated in our study as our cohort included medical subspecialties such as rheumatology and sports medicine. 

Potential modifiers for these results are suggested in the top MSK researcher data. Between endowed male and female MSK researchers, there were significant gender differences in H-index and graduation year, suggesting productivity and seniority may be contributing factors to achieving endowments; however, we are unable to account for any underlying systemic biases that may have contributed to or caused these differences. Similarly, when expanding our cohort to include heterogenous subspecialties, the seniority gap decreased, as did the differences in H-index and publication, suggesting that as more women enter and stay within the field of orthopaedic surgery, these gaps may shrink. The heterogeneity of our departmental data did not allow for graduation year as a covariable, but it is possible seniority may impact this cohort as well, which is a limitation of this data set. 

Additionally, sex-based disparities among authors of scientific publications have long been demonstrated in the literature and are particularly prevalent at earlier career stages, impacting the opportunity for women to start accruing impactful citations, which is the driver of the H-index [16-20]. This effect was demonstrated by Hoof et al., who found gender differences in H-index at the assistant professor level only, suggesting there may be a level of early research productivity that is critical to the attainment of promotion [21,20]. 

Although NIH funding data did not differ between men and women researchers, there was a difference in average NIH funding between endowed professors and non-endowed professors, suggesting increased funding begets more funding. Although seemingly gender neutral in this context, the NIH funding process differs from that of private funding, which often requires solicitation and philanthropic connections to foster alternative funding sources. A survey study of oncologists demonstrated that women are less comfortable with philanthropic solicitation compared to their male counterparts, which could impact their chances of securing an endowed chair [22]. Further, in his reflective piece published in Science, researcher Chris Hernandez reflects on the freedom and confidence that accompanied his tenure, in which stable funding afforded him the opportunity for more creative research, which, in turn, awarded him more funding, a stark contrast to the risk-averse proposals of his early career that often went unnoticed by the NIH [23]. His perspective showcases not only the risk aversion that often accompanies minority status but also the exponential effects of funding, thus highlighting how gender differences in endowments may negatively affect female orthopaedic surgeons’ ability to participate in research.

While there is limited data in the medical subspecialties pertaining to the objective effects of endowment status or the selection of awardees, Trevino et al. reflected on the constraints of their own male-dominated field of academic business management and found that after controlling for research performance and other factors, women are less likely to be awarded named professorships, particularly when the endowed chair is awarded to an internal candidate. Further, this team found that women derive lower returns from their scholarly achievements, such as an endowed chair appointment [9]. These researchers postulate that one rationale for awarding a chair to an internal candidate is the fear that a prominent candidate will be recruited by a competing institution. This seemingly gender-neutral criterion may place women at a disadvantage in advancement decisions if they are perceived as embedded in their present institution, family, and local community, while male faculty may be perceived to be less subject to these constraints. Certainly, stereotypes make an impact, but it is difficult to discern which ones are at play and when. Steinpress et al. demonstrated that researchers were more likely to choose a tenure applicant whose CV had a male name than an identical CV with a female name [10]. From a data perspective, a recent JBJS article by Ponzio et al. does validate gender differences in work-family integration in orthopaedic surgery, with 30% of female orthopaedic surgeons reporting having no children compared to 8% of men. Those women who do have children report that they have more responsibility for parenting and household duties than their male colleagues [9]. Further research is needed to discern how family responsibility or gender stereotypes may shape geographic career advancement and endowment status.

In this study, we are limited by the accuracy of publicly available data and the constraints of our study groups. We focused on the Top 100 programs and highly funded MSK researchers, and it is possible that the expansion of this group could have yielded more endowment data. Of note, institutional websites can be outdated or lack transparency, limiting the accuracy of our data collection, and estimations of gender can be incorrect and neglect alternative, non-binary definitions of gender. The heterogeneity of the data available limited our ability to accurately report graduation year for the orthopaedic department cohort, for which seniority, as it was in the MSK research group, may also be a modifier. Overall, this information was found to be largely consistent and validated by contacting endowment recipients and department chairs when discrepancies arose (less than 10 participants warranted individual contact). We also felt adherence to publicly available data was important in that this is the data that is available to applicants and the public as they form impressions of our institutions, accomplishments, and pitfalls. There may also be variables that modulate endowment status that were not examined in this study, such as race, institutional prestige, or family responsibility, as discussed above. And as discussed earlier, the H-index in isolation is not representative of productivity but a reflection of seniority or industry support [24]. Lastly, while seniority accounts for some of the differences in our results, it should not be used to discount the importance of timely action. If we are to continue our current equity goals, it has been estimated that we will not achieve 30% diversity in orthopaedic training until 2060, let alone endowed professorship and the potential financial, clinical, and recruitment effects that may accompany it [7,9].

## Conclusions

In conclusion, endowments afford awardees considerable prestige, and gender disparities at this level are significant. If we are to continue to combat the perception and actuality of our male-dominated field, we must look to achieve equity at all levels, especially in leadership positions and especially for the endowed. More research and accountability are needed to understand the effects of endowment on career advancement, research productivity, and prestige in orthopaedic surgery. However, given that much of senior leadership remains in the single digits, improvement is attainable, and it is attainable at all levels, from recruitment to selection and even in the naming of prestigious endowments in orthopaedic surgery.

## References

[1] Klyce W, Nhan DT, Dunham AM, El Dafrawy MH, Shannon C, LaPorte DM (2020). The times, they are a-changing: women entering academic orthopedics today are choosing nonpediatric fellowships at a growing rate. J Surg Educ.

[2] Van Heest AE, Agel J, Samora JB (2021). A 15-year report on the uneven distribution of women in orthopaedic surgery residency training programs in the United States. JB JS Open Access.

[3] Ramirez RN, Franklin CC (2019). Racial diversity in orthopedic surgery. Orthop Clin North Am.

[4] Bernstein J, Dicaprio MR, Mehta S (2004). The relationship between required medical school instruction in musculoskeletal medicine and application rates to orthopaedic surgery residency programs. J Bone Joint Surg Am.

[5] O'Connor MI (2016). Medical school experiences shape women students' interest in orthopaedic surgery. Clin Orthop Relat Res.

[6] Bi AS, Fisher ND, Bletnitsky N, Rao N, Egol KA, Karamitopoulos M (2022). Representation of women in academic orthopaedic leadership: where are we now?. Clin Orthop Relat Res.

[7] Harrington MA, Rankin EA, Ladd AL, Mason BS (2019). The orthopaedic workforce is not as diverse as the population it serves: where are the minorities and the women? AOA critical issues symposium. J Bone Joint Surg Am.

[8] Kroin E, Garbarski D, Shimomura A, Romano J, Schiff A, Wu K (2019). Gender differences in program factors important to applicants when evaluating orthopaedic surgery residency programs. J Grad Med Educ.

[9] Treviño LJ, Gomez-Mejia LR, Balkin DB, Mixon FG Jr (2018). Meritocracies or masculinities? The differential allocation of named professorships by gender in the academy. J Manage.

[10] Steinpreis RE, Anders KA, Ritzke D (1999). The impact of gender on the review of the curricula vitae of job applicants and tenure candidates: a national empirical study. Sex Roles.

[11] Chambers CC, Ihnow SB, Monroe EJ, Suleiman LI (2018). Women in orthopaedic surgery: population trends in trainees and practicing surgeons. J Bone Joint Surg Am.

[12] Saxena S, Cannada LK, Weiss JM (2020). Does the proportion of women in orthopaedic leadership roles reflect the gender composition of specialty societies?. Clin Orthop Relat Res.

[13] Sobel AD, Cox RM, Ashinsky B, Eberson CP, Mulcahey MK (2018). Analysis of factors related to the sex diversity of orthopaedic residency programs in the United States. J Bone Joint Surg Am.

[14] Kimball BA, Johnson BA (2012). The inception of the meaning and significance of endowment in American Higher Education, 1890-1930. Tea Col Rec.

[15] Gold JA, Roubinov D, Jia LS, Griffith KA, Carethers JM, Mangurian C, Jagsi R (2020). Gender differences in endowed chairs in medicine at top schools. JAMA Intern Med.

[16] Bastian S, Ippolito JA, Lopez SA, Eloy JA, Beebe KS (2017). The use of the h-index in academic orthopaedic surgery. J Bone Joint Surg Am.

[17] Hoof MA, Sommi C, Meyer LE, Bird ML, Brown SM, Mulcahey MK (2020). Gender-related differences in research productivity, position, and advancement among academic orthopaedic faculty within the United States. J Am Acad Orthop Surg.

[18] Jagsi R, Guancial EA, Worobey CC The “gender gap” in authorship of academic medical literature: a 35-year perspective. N Engl J Med.

[19] Larson AR (2021). Implications of sex disparities in the h-index for academic medicine. JAMA Netw Open.

[20] Reed DA, Enders F, Lindor R, McClees M, Lindor KD (2011). Gender differences in academic productivity and leadership appointments of physicians throughout academic careers. Acad Med.

[21] Xu RF, Varady NH, Chen AF, Earp BE (2022). Gender disparity trends in authorship of hand surgery research. J Hand Surg Am.

[22] Walter JK, Griffith KA, Jagsi R (2015). Oncologists’ experiences and attitudes about their role in philanthropy and soliciting donations from grateful patients. J Clin Oncol.

[23] Hernandez C (2021). By playing it safe, I became a Latino scientist but that approach held me back. Science.

[24] Boddapati V, Sachdev R, Fu MC, Camp CL, Marx RG, Dines JS (2018). Increasing industry support is associated with higher research productivity in orthopaedic surgery. J Bone Joint Surg Am.

[25] (2024). Health News US: Best Hospital Rankings. https://health.usnews.com/best-hospitals/rankings/orthopedics,%C2%A0https://www.doximity.com/residency/programs?specialtyKey=bd234238-6960-4260-9475-1fa18f58f092-orthopaedic-surgery&sortByKey=research_rank&trainingEnvironmentKey=&intendedFellowshipKey=].

[26] (2024). BRIMR: Rankings of NIH funding in 2021. https://brimr.org/brimr-rankings-of-nih-funding-in-2021.

